# COVID-19 uncertainty and sleep: the roles of perceived stress and intolerance of uncertainty during the early stage of the COVID-19 outbreak

**DOI:** 10.1186/s12888-021-03310-2

**Published:** 2021-06-14

**Authors:** Dan Wu, Tingzhong Yang, Daniel L. Hall, Guihua Jiao, Lixin Huang, Can Jiao

**Affiliations:** 1 School of Psychology, Shenzhen University, Shenzhen, 518060 Guangdong China; 2 Women’ s Hospital/Center for Tobacco Control Research, Zhejiang University School of Medicine, Hangzhou, 310058 Zhejiang China; 3 Massachusetts General Hospital, Harvard Medical School, Boston, MA 02114 USA; 4 Department of Psychology/Research Center on Quality of Life and Applied Psychology, Guangdong Medical University, Dongguan, 523808 Guangdong China

**Keywords:** Uncertainty about COVID-19, Intolerance of uncertainty, Perceived stress, Poor sleep

## Abstract

**Background:**

The COVID-19 pandemic brings unprecedented uncertainty and stress. This study aimed to characterize general sleep status among Chinese residents during the early stage of the outbreak and to explore the network relationship among COVID-19 uncertainty, intolerance of uncertainty, perceived stress, and sleep status.

**Methods:**

A cross-sectional correlational survey was conducted online. A total of 2534 Chinese residents were surveyed from 30 provinces, municipalities, autonomous regions of China and regions abroad during the period from February 7 to 14, 2020, the third week of lockdown. Final valid data from 2215 participants were analyzed. Self-report measures assessed uncertainty about COVID-19, intolerance of uncertainty, perceived stress, and general sleep status. Serial mediation analysis using the bootstrapping method and path analysis were applied to test the mediation role of intolerance of uncertainty and perceived stress in the relationship between uncertainty about COVID-19 and sleep status.

**Results:**

The total score of sleep status was 4.82 (SD = 2.72). Age, place of residence, ethnicity, marital status, infection, and quarantine status were all significantly associated with general sleep status. Approximately half of participants (47.1%) reported going to bed after 12:00 am, 23.0% took 30 min or longer to fall asleep, and 30.3% slept a total of 7 h or less. Higher uncertainty about COVID-19 was significantly positively correlated with higher intolerance of uncertainty (r = 0.506, *p* < 0.001). The mediation analysis found a mediating role of perceived stress in the relationship between COVID-19 uncertainty and general sleep status (*β =* 0.015, 95%C.I. = 0.009–0.021). However, IU was not a significant mediator of the relationship between COVID-19 uncertainty and sleep (*β =* 0.009, 95%C.I. = − 0.002–0.020). Moreover, results from the path analysis further showed uncertainty about COVID-19 had a weak direct effect on poor sleep (β = 0.043, *p* < 0.05); however, there was a robust indirect effect on poor sleep through intolerance of uncertainty and perceived stress.

**Conclusions:**

These findings suggest that intolerance of uncertainty and perceived stress are critical factors in the relationship between COVID-19 uncertainty and sleep outcomes. Results are discussed in the context of the COVID-19 pandemic, and practical policy implications are also provided.

**Supplementary Information:**

The online version contains supplementary material available at 10.1186/s12888-021-03310-2.

## Introduction

The World Health Organization’s (WHO’s) China Country Office was informed of cases of pneumonia of unknown cause detected in Wuhan City, Hubei Province, on December 31, 2019 [1]. On January 23, in response to the growing COVID-19 epidemic, the Chinese government locked down Wuhan City [2]. During the first 14 days of lockdown, confirmed cases in China increased dramatically from 571 on January 23 to 28,060 on February 6 [1]. Due to the enforcement of the lockdown, most people were restricted to their homes. This confinement has resulted in a changed lifestyle, disrupted chronobiological rhythms, and impacted on mental health, including sleep issues [3–5]. An online survey demonstrated that 30% of participants among Wuhan residents had a sleep disorder during the incipient pandemic [6]. Similarly, an Italian study reported 42.2% of the sample had sleep disturbances and, among them, 17.4% reported moderate/severe insomnia in Italy, which was the first European country to enter a nationwide lockdown in 2020 [4]. A systematic review and meta-analysis demonstrated a global pooled prevalence rate of sleep problems among all populations was 35.7% (95% C.I.:29.4–42.4%) which suggested the sleep problem was a major public health issue during the COVID-19 pandemic [5]. Sleep is an integral part of proper human function [7]. When facing COVID-19, sleep becomes essential because of its benefits for mental and physical health [5]. Poorer sleep quality is associated with increased susceptibility to viral infections, lower cognitive functioning, poorer job performance, and worsened mental health [8]. Understanding the influencing mechanism of sleep during the COVID-19 pandemic may help the residents to maintain good sleep status and provide support for the design and implementation of interventions for sleep health in pandemic conditions.

When confronted with such a sudden and actual disaster, people realize that uncertainty surrounds every aspect of life [9]. Uncertainty is one of the major cognitive and psychological stressors [10]. Further, factors relating to stress are one of the most important concomitants of sleep complaints in the general population [3, 11]. The concept of uncertainty in illness was defined as “the inability to determine the meaning of illness-related events” [12]. Applicable to COVID-19, uncertainty according to Mishel’s initial definition is a cognitive state that occurs when the decision maker is unable to assign definite values to objects and events and/or is unable to accurately predict outcomes because of a lack of sufficient cues [12]. Since this unexpected COVID-19 pandemic has swept across the world, limited knowledge of COVID-19 for diagnosis and treatment, unpredictability of the natural course of contagious illness, and gross disruption of societal functioning and people’s routines may all represent potential sources of uncertainty. Uncertainty has been identified as the greatest single psychological stressor for patients with a life-threatening illness [13]. Given the sustained uncertainties and challenges in managing COVID-19, it is likely that, if unmanaged, COVID-19 related uncertainty may persist and continue to impact sleep outcomes.

Intolerance of uncertainty (IU) relates to the cognitive unacceptability of uncertainty and represents a dispositional characteristic of those people who are more likely to “find ambiguity stressful and upsetting, believe uncertainty is negative and should be avoided, and have difficulty functioning in uncertain situations” [14, 15]. IU was first defined by Freeston and colleagues as “a relatively broad construct representing cognitive, emotional, and behavioral reactions to uncertainty in everyday life situations” [16]. Carleton further explicitly delineated the definition of IU that includes the triggering stimuli, the response, and the incapacity to endure the associated perception of uncertainty [17, 18]. Although uncertainty is pervasive and inherent in people’s daily lives, individuals who have a high degree of perceived uncertainty or who are high in IU experience might significantly impair their daily functioning [18–20]. A substantial body of research in the context of both normal time and COVID-19 pandemic suggests that individual differences in IU foster stress, depression, and anxiety [19–22]. Several studies have uncovered the mutual relationship between IU and fear of COVID-19 [21, 23]. Disease fear increased IU, and higher IU aggravated fear of COVID-19, subsequently weakening people’s well-being [23]. Theoretical conceptualizations posit that those high in IU are likely to rely on maladaptive behaviors as a coping mechanism when faced with uncertain and potentially aversive situations [24].

A meta-analysis shows that the association between IU and symptoms was observed across all kinds of mental disorders [25]. A significant association between IU and sleep disturbances in young adults and more specifically Iranian adults was examined [26]. A large body of evidence from clinical research has demonstrated that generalized anxiety disorder patients with greater IU suffer from sleep dysfunctions including longer sleep latency, decreased sleep duration, decreased total sleep efficiency, and increased waking periods during sleep time [27, 28]. Research by our group has also demonstrated strong associations between cancer-related uncertainty and insomnia severity among cancer survivors [29]. There is a plethora of studies on uncertainty for clinical patients with chronic diseases. However, research on uncertainty in a lethal and urgent infectious disease outbreak and its side effects is limited among non-patients [25, 30]. The role of IU in sleep problems has not been studied extensively in the context of the COVID-19 pandemic [5, 15]. Unknown is how IU impacts the public in cognitively processing a COVID-19-related stressor, as well as how their sleeping behaviors are influenced by such serious outbreak-related events.

Based on previous literature, IU appears to be an important element of cognitive vulnerability affecting sleep quality [28]. The current study seeks to explore the potential mechanism for influencing sleep status during the early stage of COVID-19 outbreak, and to examine how IU and perceived stress impact the relations between uncertainty about COVID-19 and sleep in a sample of Chinese general residents. The following four hypotheses and research questions were evaluated in this study. First, we expected a positive association between perceived uncertainty about COVID-19 and IU and, second, following prior studies, an association between IU and perceived stress [19, 31]. Third, in line with previous research [32, 33], we predicted a positive association between stress and poor sleep. Finally, we treat as exploratory the possibility that IU and stress would play a mediating role in the relationship between uncertainty about COVID-19 and poor sleep.

## Methods

### Study design and participants

A cross-sectional correlational design and a combination of convenience and snowball sampling were utilized in this study. The online survey was conducted from February 7 to 14, 2020 (the third week after the lockdown of Wuhan City) based on the Wenjuanxing Platform (https://www.wjx.cn/app/survey.aspx) Twenty psychology students were trained as research assistants to recruit participants through WeChat and other major Chinese social networking platforms. Our sample covered 30 provinces, municipalities, autonomous regions of China, and regions abroad. A total of 2654 potential participants were contacted online, and 2534 agreed to participate in the survey. A more detailed description of the survey and the data can be found in Wu et al. [34]. Participants took approximately 15-min to complete the questionnaire. The study protocol was approved by the Ethics Committee of Shenzhen University, and written consent was obtained from all participants prior to administration of the questionnaire.

### Measures

The survey questionnaire covered five categories: (a) demographics, (b) sleep status, (c) uncertainty about COVID-19, (d) intolerance of uncertainty, and (e) perceived stress.

### Dependent variables

#### General sleep status

General sleep status was measured by the 6-item Sleep Questionnaire regarding the Pittsburgh Sleep Quality Index (PSQI) [35]. The participants were asked about their sleep status during the past month by the following questions 1) how would you rate your sleep quality overall; 2) when have you usually gone to bed at night; 3) how long (in minutes) has it usually take to fall asleep; 4) how many hours of actual sleep did you get at night; 5) how often have you taken medicine to help you sleep; 6) how often have you had trouble staying awake [24]. Subjective sleep quality, sleep bedtime, sleep latency, sleep duration, use of sleeping medication, and daytime dysfunction were assessed. Each item is divided into four levels with a 4-point Likert scale. For subjective sleep quality, the responses were coded as 0 = very good, 1 = good, 2 = bad, and 3 = very bad. The answers for sleep bedtime were coded as 0 = before 11:00 pm, 1 = 11:00 pm-12:00 am, 2 = 12:00 am-1:00 am, and 3 = after 1:00 am. The options for sleep latency were scored as 0 = less than 15 min, 1 = 16–30 min, 2 = 31–60 min, and 3 = more than 60 min. The answers for sleep duration were scored as 0 = more than 7 h, 1 = 6–7 h, 2 = 5–6 h, and 3 = less than 5 h. The responses for use of sleeping medication and daytime dysfunction were all assigned as 0 = none, 1 = less than once a week, 2 = once or twice a week, and 3 = three or more times a week. When we factor-analyzed the sleep questionnaire in our study, we divided the components into two factors, which accounted for 52.75% of the variance. The components of sleep duration and use of sleep medication were more closely associated, and the other components were more closely associated in our subjects. The six-component scores were rated on a 0–3 scale and then summed to yield a global sleep score which has a range of 0–18, with higher scores reflecting poorer sleep quality. The total score of sleep status in this study has been found to have a good correlation (r = 0.699, *p* < 0.01) with the single item of subjective sleep quality of the Pittsburgh Sleep Quality Index. The Cronbach’s alpha coefficient of this sleep questionnaire was 0.713, suggesting that its internal consistency was acceptable.

### Independent variables

#### Demographic characteristics

The following sociodemographic information was collected during the survey: date of birth, gender, place of residence, ethnicity, marital status, educational attainment, occupation, and per capita annual family income. Specific experience with COVID-19, including infection and quarantine status, were also measured [34].

#### Uncertainty about COVID-19

The 10-item Uncertainty about COVID-19 Scale was developed with reference to the Mishel Uncertainty in Illness Scale [12]. We applied a 5-point Likert-type scale ranging from strongly agree to strongly disagree in tapping public perception of uncertainty about the COVID-19 outbreak [34]. Item scores were summated to obtain a total uncertainty score. The higher the score, the greater the perceived uncertainty about the disease. The Kaiser-Meyer-Olkin measure of sampling adequacy was 0.866, and Bartlett’s test of sphericity was significant (*P* < 0.001), suggesting the sample was factorable. The factor loading on each item exceeded 0.5. Finally, two distinct factors of “lack of information and clarity” and “unpredictability” were extracted, which accounted for 59% of the variance. The Cronbach’s alphas coefficient was 0.75 for lack of information and clarity, and 0.85 for unpredictably, respectively. The reliability coefficient for the Uncertainty about COVID-19 Scale was 0.85, suggesting good reliability.

#### Intolerance of uncertainty

Intolerance of Uncertainty was measured by the 12-item Intolerance of Uncertainty Scale (IUS-12) [36]. The IUS-12 was a widely used and reliable psychometric instrument that consisted of a stable two-factor structure, representing anxious and avoidance components of intolerance of uncertainty [36, 37]. Items are scored on a Likert scale ranging from 1 (not at all characteristic of me) to 5 (entirely characteristic of me), yielding possible total scores from 12 to 60. Higher overall scores correspond to higher intolerance of uncertainty. The Cronbach’s α coefficient for the Intolerance of Uncertainty Scale in this sample was 0.89.

#### Perceived stress

Stress was measured by the Perceived Stress Scale, Chinese version (CPSS) which has good reliability and validity and has been widely used to assess perceived stress in community settings across China [38, 39]. This scale comprised 14 items that assessed the perception of stress during the month prior to the survey. Items were rated on a 5-point Likert-type scale and ranged from 1 (never) to 5 (very often). Item scores were summed to yield a total stress score. The higher the total score, the greater the perceived level of stress [38, 39]. The internal reliability of the CPSS in this sample, measured by Cronbach’s α, was 0.77.

### Data analysis

All survey data were entered into a Microsoft Excel database, and then imported into SPSS (version 22.0) and Amos 21.0 for statistical analysis. Descriptive statistics and univariate analysis on sleep status were conducted. Cronbach’s alpha coefficient and exploratory factor analysis were used to examine the reliability and validity of the sleep questionnaire, Uncertainty about COVID-19 Scale, Perceived Stress Scale, and Intolerance of Uncertainty Scale. Pearson correlational analysis was applied to explore the relationships among intolerance of uncertainty, uncertainty about COVID-19, perceived stress, and sleep status. Mediation analysis using the bootstrapping method was performed with PROCESS. Regards to the mediation model, the dependent variable was general sleep status, and uncertainty about COVID-19 was the independent variable. The mediators were IU and perceived stress, and the covariates were the significant demographic characteristics, infection, and quarantine status via univariate analysis. In the present study, the 95% CI of the total effect, direct effect, and indirect effects was obtained with 5000 bootstrap resamples. A significant indirect effect via mediators between dependent and independent variables was identified if the 95% CI does not contain zero [21]. Hypotheses were also tested by Structural Equation Modeling (SEM) with maximum likelihood estimation. SEM is a multivariate statistical analysis technique that is used to analyze structural relationships, including causal modeling, simultaneous equation modeling, path analysis, and so on [40]. This study used the path analysis to examine the network relationship among uncertainty about COVID-19, intolerance of uncertainty, perceived stress, and poor sleep. Finally, the model modification was also conducted to adjust the estimated model by adding or removing the path parameters [41]. Five of the most popular fit indices were selected to evaluate the overall fit of the model [40, 41]. These fit indices were a Chi-square to degrees of freedom ratio (χ2/df), the goodness-of-fit index (GFI), the comparative fit index (CFI), the root mean square error of approximation (RMSEA), standardized root mean square residual (SRMR) [41].

## Results

The response rate could not be calculated because the study population was a convenience sample. A total of 2534 participants were surveyed, of whom 2215 (87.4%) completed valid questionnaires. Among this general public sample, 54 respondents reported that they were infected by COIVD-19 and the remaining 2161 participants stated that they were uninfected. Over two-thirds of the participants were female (67.2%) and urban residents (68.7%), 75.3% were single (75.3%), and 50.2% were aged 20 to 24 years. The characteristics of the respondents are summarized in Table 1. The statistically significant differences in sociodemographic characteristics for total sleep scores were for age, place of residence, ethnicity, and marital status. Individual infection status and their friends, relatives, colleagues, and neighbors’ quarantine status due to infection or suspected infection by COVID-19 were all significantly correlated with sleep status.

**Table 1:** Demographic characteristics and total score of general sleep status

Variables	N	%	Mean (SD)	F/t	p
Age				6.800	< 0.001**
< 20	411	18.6	4.49 (2.64)		
20–24	1113	50.2	5.00 (2.77)		
25–29	240	10.8	5.28 (2.74)		
30–39	209	9.4	4.55 (2.66)		
40+	242	10.9	4.36 (2.53)		
Gender				0.235	0.628
Male	726	32.8	4.86 (2.80)		
Female	1489	67.2	4.80 (2.68)		
Place of residence				12.910	< 0.001**
Urban	1522	68.7	4.96 (2.74)		
Rural	683	31.3	4.52 (2.64)		
Ethnicity				8.269	0.004**
Han	2155	97.3	4.79 (2.71)		
Minority	60	2.7	5.82 (2.87)		
Marital status				4.253	0.014*
Unmarried	1699	75.3	4.88 (2.73)		
Married	517	23.3	4.57 (2.67)		
Divorced/widowed	29	1.3	5.72 (2.52)		
Education				2.075	0.101
Junior high school or less	196	8.8	4.68 (2.82)		
High school	233	10.5	5.04 (2.86)		
Junior college	263	11.9	5.13 (3.02)		
College or higher	1523	68.8	4.76 (2.63)		
Occupation				0.621	0.648
Public official /professionals	257	11.6	4.71 (2.69)		
Enterprise personnel	238	10.7	4.91 (2.90)		
Commerce/service/operations	215	9.7	5.03 (2.61)		
Students	1311	59.2	4.78 (2.70)		
Others	194	8.8	4.91 (2.80)		
Household annual income (RMB)				0.750	0.522
Less than 20,000	475	21.4	4.78 (2.70)		
20,000-60,000	832	37.6	4.74 (2.67)		
60,000-100,000	516	23.3	4.90 (2.74)		
More than 100,000	392	17.7	4.95 (2.82)		
Infected by COVID-19				34.80	< 0.001**
Yes	54	2.4	6.96 (2.15)		
No	2161	96.5	4.77 (2.71)		
Friends/colleagues/relatives quarantined due to COVID-19				15.91	< 0.001**
Yes	132	6.0	5.73 (2.64)		
No	2083	94.0	4.76 (4.72)		
Neighborhood quarantined due to COVID-19				24.41	< 0.001**
Yes	278	12.6	5.57 (2.67)		
No	1937	87.4	4.71 (2.71)		

* < 0.05; ** < 0.01

Table 2 demonstrated the sleep status of participants during the early stage of the COVID-19 outbreak. Among the total sample, 47.1% went to bed after 12:00 am, 23.0% took more than 30 min to fall asleep, and 30.3% of participants’ sleep duration was less than 7 h. A minority of participants (8.5%) used sleeping medication and 20.9% rated their sleep quality bad or very bad. Half of the participants experienced daytime dysfunction.

**Table 2:** The sleep status of participants during the early stage of COVID-19 outbreak

Variables	n	%	Variables	n	%
Bedtime			Subjective Sleep quality		
Before 11:00 pm	550	24.8	Very good	527	23.8
11:00 pm-12:00 am	622	28.1	Good	1226	55.3
12:00 am-1:00 am	589	26.6	Bad	411	18.6
After 1:00 am	454	20.5	Very Bad	51	2.3
Sleep latency			Use of sleeping medication		
≤ 15 mins	855	38.6	None	2026	91.5
16–30 min	851	38.4	< 1 /per week	90	4.1
31–60 min	284	12.8	1–2 /per week	81	3.7
≥ 60 mins	225	10.2	≥3 / per week	18	0.8
Sleep duration			Daytime dysfunction		
< 5 h	58	2.6	None	1108	50.0
5–6 h	187	8.4	< 1 /per week	515	23.3
6–7 h	426	19.2	1–2 /per week	347	15.7
≥ 7 h	1544	69.7	≥3 / per week	245	11.1

The descriptive statistics and bivariate correlations were displayed in Table 3. The mean total score of sleep status was 4.82 (95% C.I. 4.71–4.94). Each item score for intolerance of uncertainty, uncertainty about COVID-19, and perceived stress were 3.03 (95% C.I. 3.00–3.06), 3.08 (95% C.I. 3.05–3.10), and 2.92 (95% C.I. 2.90–2.94), respectively. The correlation between intolerance of uncertainty and uncertainty about COVID-19 (r = 0.506, *p* < 0.001) and perceived stress and poor sleep (r = 0.336, p < 0.001) were positive. Meanwhile, intolerance of uncertainty (r = 0.538, p < 0.001) and uncertainty about COVID-19 (r = 0.360, p < 0.001) also positively related to perceived stress.

**Table 3:** Inter-correlations and descriptive statistics of study variables

	Variables					Total Score		Item Score	
		1	2	3	4	M (SD)	95% C.I.	M (SD)	95% C.I.
1	Intolerance of uncertainty	1.00				36.40 (8.19)	36.06–36.74	3.03 (0.68)	3.00–3.06
2	Uncertainty about COVID-19	0.506**	1.00			30.77 (6.47)	30.50–31.04	3.08 (0.65)	3.05–3.10
3	Perceived stress	0.538**	0.360**	1.00		40.87 (6.81)	40.58–41.15	2.92 (0.49)	2.90–2.94
4	General sleep status	0.218**	0.159**	0.336**	1.00	4.82 (2.72)	4.71–4.94	/	/

** < 0.01

The findings from serial multiple mediation analysis using the bootstrapping method were presented in Tables 4 and 5. The total effect of COVID-19 uncertainty on sleep was significant (*β =* 0.067, 95%C.I. = 0.050–0.084) after adjusting potential covariates including demographic characteristics. The results of the mediation analysis confirmed the mediating role of perceived stress in the relationship between COVID-19 uncertainty and general sleep status (*β =* 0.015, 95%C.I. = 0.009–0.021). However, IU was not a significant mediator of the relationship between COVID-19 uncertainty and sleep (*β =* 0.009, 95%C.I. = − 0.002–0.020).

**Table 4:** The results from mediation analysis using a bootstrapping method for general sleep status

Dependent variable	Independent Variable#	Beta coefficient	SE	t	p	R2	p
IU	*Constant*	19.434	2.54	7.64	< 0.001**	0.26	< 0.001**
	Uncertainty about COVID-19	0.642	0.02	27.48			
Perceived stress	*Constant*	24.991	2.07	12.09	< 0.001**	0.31	< 0.001**
	IU	0.397	0.02	23.25	< 0.001**		
	Uncertainty about COVID-19	0.125	0.02	5.75	< 0.001**		
General sleep status	*Constant*	3.723	0.95	3.92	< 0.001**	0.14	< 0.001**
	Perceived stress	0.119	0.01	12.57	< 0.001**		
	IU	0.014	0.01	1.61	0.11		
	Uncertainty about COVID-19	0.013	0.01	1.33	0.19		

# All models were adjusted for infection, quarantine status, and demographic characteristics, such as age, ethnicity, residence, marriage. Bootstrapping = 5000; * < 0.05; ** < 0.01

**Table 5:** Indirect effect of uncertainty about COVID-19 on sleep status via intolerance of uncertainty and perceived stress

Path	Coefficient	95% confidence interval	
		Boot lower limit	Boot upper limit
Uncertainty about COVID-2019 → IU → Sleep	0.009	−0.002	0.020
Uncertainty about COVID-2019 → Perceived stress→Sleep	0.015	0.009	0.021
Uncertainty about COVID-2019 → IU → Perceived stress→Sleep	0.030	0.024	0.037
Total effect	0.067	0.050	0.084
Direct effect	0.013	−0.006	0.032
Total indirect effect	0.054	0.043	0.066

**Fig. 1:**
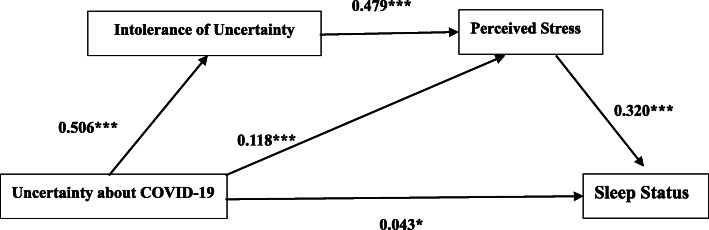
Path analysis for Uncertainty-Stress-Sleep Model during the early stage of COVID-19 outbreak. * < 0.05; ** < 0.01; *** < 0.001; Values shown are standardized coefficients. (*χ2/df* = 2.377, GFI = 0.999, CFI = 0.999, RMSEA = 0.025,SRMR = 0.008)

Based on the results of mediation analysis and previous related literature, the path analysis was also conducted to assess the hypothesized network relationships among the variables. With adding the path from uncertainty about COVID-19 to general sleep status, the modified model in Fig. 1 was fitted the data better across the whole samples (χ2/df = 2.377, CFI = 0.999, GFI = 0.999, RMSEA = 0.025, SRMR = 0.008). Specifically, uncertainty about COVID-19 had a weak direct effect on poor sleep (*β =* 0.043, *p* < 0.05). Uncertainty about COVID-19 had an indirect effect on poor sleep through intolerance of uncertainty (*β =* 0.506, *p* < 0.001) and perceived stress (*β =* 0.479, p < 0.001). Further, uncertainty about COVID-19 also had another indirect pathway impacting poor sleep via perceived stress (*β =* 0.118, p < 0.001).

## Discussion

The COVID-19 brings unprecedented uncertainty to individuals and society at the initial stage of the outbreak. Uncertainty is an ever-present feature when people face the COVID-19 outbreak. This study investigated the general sleep status of Chinese residents during the third week of lockdown and identified the potential risk factors for influencing sleep from the perspective of uncertainty. We found urban residents, the minority, and the divorced/widowed people had a higher total score of sleep status, suggesting they experienced worse sleep. The government should pay more attention to these populations and their sleep health issues during the COVID-19 pandemic. Participants who were infected by COVID-19 and whose friends, relatives, colleagues, or neighbors were quarantined due to infection or suspected infection by COVID-19 had a significantly higher sleep score than their counterparts. This was in line with the results from other studies showing patients infected with COVID-19 appeared to be the most affected group with a prevalence of sleep problems [5].

Our results from path analysis showed that this kind of uncertainty caused by COVID-19 might have influenced the public’s sleeping behaviors and sleep quality. The Illness Uncertainty Theory explains how patients cognitively process an illness-related stimulus, as well as how they structure the meaning of such an event, and proposes that high uncertainty is associated with diminished capacity to process new information, predict outcomes, and adapt to the illness [42]. Not only the illness uncertainty and fear of disease progression affect psychosocial adjustment and the quality of life [43], but the uncertain social situation caused by COVID-19 would also disrupt the people’s routine sleep schedule. A recent study revealed that uncertainty about COVID-19 was negatively correlated with general response behaviors including eating and exercise [34]. The possible mechanism was that COVID-19 related uncertainty would cause cognitive confusion, exhaust an individual’s energy, diminish perceived control, and divert attention from routine healthy behaviors which might affect regular sleep patterns as well [34, 44].

However, we found an effect size smaller than expected for the correlation between uncertainty about COVID-19 and poor sleep status. For possible potential explanation, its key mechanism of the relationship between uncertainty about COVID-19 and poor sleep might be mediated by intolerance of uncertainty and corresponding stress indirectly. The current study revealed higher uncertainty about COVID-19 was significantly positively correlated with higher intolerance of uncertainty, which further increased perceived stress, and subsequently weakened sleep status. COVID-19 is a new virus, related information is unknown or imperfect, and there is much ambiguity surrounding the disease [44]. A host of evidence supports that uncertainty is even more intolerable than knowing the inevitability of something bad happening [44, 45]. Uncertainty about COVID-19 involved both disease uncertainty and unpredictability for returning to normal social life which might results in a perceived fear and intolerance [21]. This high correlation between COVID-19 uncertainty and IU may also be explained by a risk-averse and certainty-oriented culture [34]. Chinese people tend to take uncertainty about COVID-19 as a continuous threat and then become more intolerable with it.

Along with the previous studies, our study has found a positive correlation between IU and perceived stress [19, 21, 31, 46]. The link between uncertainty and stress theoretically suggested that IU could increase the negative impact of stressors [31, 47]. The relationship between IU and psychological distress was also examined among the general population during the first wave of the COVID-19 pandemic in the United Kingdom [22]. IU was predictive of mental health difficulties such as depression, anxiety, and fear and this was also mediated by their coping responses [22]. Those mental health difficulties were highly associated with stress and maladaptive coping would, in turn, exacerbate the perceived stress.

In consistent with prior sleep researches, it has been shown that stress uniquely predicts poor sleep quality regardless of whether it is related to COVID-19 or not [28, 33]. A Chinese online survey conducted from February 18 to 25, 2020 which was close to the date of our investigation found that around one-third of participants were poor sleepers and further indicated perceived stress affected sleep quality through anxiety [33]. Based on Stress and Coping Theory, people perceived stress when taking COVID-19 related events as an environmental stimulus which results in a changed emotional stage such as anxiety that, in turn, affects one’s health including sleep quality [33]. Furthermore, previous research has overlooked the role of cognitive dispositions that might exacerbate stress, thus contributing to the continuation of sleep problems [15]. Perceived uncertainty and IU might be such dispositions.

Nevertheless, it is worth noting that IU was not correlated with sleep directly. To our knowledge, IU has been less studied with sleep problems [28] and only limited studies examined IU as antecedents of sleep problems. One study showed IU was strongly associated with anxiety sensitivity, in turn influencing both insomnia severity and sleep quality via depression and anxiety [15]. IU might be involved in the process that links personality to sleep problems as well [15]. The Uncertainty and Anticipation Model of Anxiety identified that individual differences in IU are related to physiological indicators of responses to uncertainty, that are associated with increased risk for anxiety [48, 49]. Another study demonstrated that worry partially mediated the relationship between IU and poor sleep quality in an adolescent sample [28]. In our study, perceived stress mediated the indirect effect of IU on sleep status in a general community sample.

Another pathway of COVID-19 uncertainty influencing sleep in this study was mediated by perceived stress. A panel study showed uncertainty stress was positively associated with disease fear, and negatively associated with self-efficacy, and prevention behaviors, which might lead to irregular sleep status as maladaptive coping strategies [44]. Our study has confirmed perceived stress is a strong factor affecting sleep status. Given the great impact of the individual ability to tolerate uncertainty on the residents’ level of perceived stress, it would be worth paying particular attention to such a skill when facing the COVID-19. The practical implications of the present study exhibited that it would be crucial to introduce or to empower tools and strategies that could increase residents’ ability to tolerate uncertainty and cope with related stress, further to improve the sleep status. In addition, interventions on the public’s training and education to have better access to information about COVID19 and being able to process it adequately may be a possible and alternative strategy for increasing sleep quality. Recently, work by our group has focused on disseminating mind-body resiliency skills aimed at reducing intolerance of uncertainty and improving stress management skills using synchronous, virtual platforms that can be accessed while engaging in social distancing [50]. Among cancer survivors, another population facing illness uncertainty, training in relaxation, meditation, cognitive-behavioral coping techniques, and positive psychology skills (e.g., gratitude, using humor) have been associated with favorable improvements in tolerance of uncertainty [51]. Similar resiliency training approaches may thus yield downstream benefits on improving sleep during the COVID-19 pandemic, although to the best of our knowledge this remains unexamined.

### Strengths and limitations

This study has several strengths and limitations. First, general sleep status was assessed via self-report, which may be susceptible to recall bias. Still, the Pittsburgh Sleep Quality Index is psychometrically strong, and objective sleep measures (e.g., actigraphy) have been critiqued for not accounting for perceived sleep disturbances. Second, this report included a large sample of over 2500 Chinese residents. While the cross-sectional analyses precluded our ability to draw causal conclusions, our use of structural equation modeling capitalized on the large sample size to test multiple indirect pathways from COVID-19 uncertainty to sleep quality. Third, while we were able to collect surveys during the early stages of the pandemic, we recognized that the over-representation of college students and participants from Guangdong Province suggests sampling bias in this sample which may not generalize to the whole Chinese population, as well as the experiences of uncertainty in other nations whose governments responded differently to managing COVID-19. Finally, the study did not include a measure of mental health problems or specific sleep diseases such as insomnia. Inclusion might have permitted examination of the impact of COVID-19 uncertainty and IU on both overall sleep status and specific sleep-related disorder, after controlling for some other important psychological and behavioral factors.

## Conclusions

The current study suggested that the Chinese residents’ uncertainty about COVID-19 is directly associated with poor sleep but also indirectly correlated through the mediating role of IU and perceived stress. This study might provide evidence-based information to the governments and policymakers to design effective health intervention strategies targeted at reducing the COVID-19 uncertainty, improvements in tolerance of uncertainty and coping with related stress, further to improve the sleep status during the stage of COVID-19 pandemic.

## Supplementary Information

**Additional file 1.**

## Data Availability

Data available on request from the corresponding author.
