# Supplementary material for: COVID-19 uncertainty and sleep: the roles of perceived stress and intolerance of uncertainty during the early stage of the COVID-19 outbreak
Source: BMC Psychiatry. 2021 Jun 14;21:306. doi: 10.1186/s12888-021-03310-2 (PMC8200549; doi:10.1186/s12888-021-03310-2)
Supplement: Supplementary file 1 — Additional file 1. [file 12888_2021_3310_MOESM1_ESM.docx]

**Appendix: The items for Uncertainty about COVID-19 Scale**

1. I have many unanswered questions about COVID-19.
2. I am unsure how to deal with a fever, cough or other symptoms.
3. The effectiveness of the treatments or medications COVID-19 patients are receiving is undetermined.
4. The transmission route of COVID-19 is unclear.
5. There are so many different opinions about the prognosis of COVID-19.
6. Because of the unpredictability of the COVID-19 outbreak, I cannot plan for the future.
7. Because of the COVID-19 outbreak, what I can do and cannot do is in flux.
8. I am unsure when the COVID-19 outbreak will end.
9. I am unsure when the social normal order will return.
10. It is unclear what is going to happen to me.
